# Research trends and hotspots in exercise rehabilitation for coronary heart disease: A bibliometric analysis

**DOI:** 10.1097/MD.0000000000036511

**Published:** 2023-12-15

**Authors:** Qing Wen, Qun-Hua Ma, Lin-Zhang Li, Xue-Wu Song, Hu-Kui Han, Gui-Yu Huang, Xiao-Li Tang

**Affiliations:** a Department of Cardiovascular Internal Medicine 1, Sichuan Provincial People’s Hospital, University of Electronic Science and Technology of China, Chengdu, China; b Chinese Academy of Sciences Sichuan Translational Medicine Research Hospital, Chengdu, China; c Comprehensive care unit, Chengdu Wen jiang District People’s Hospital, Chengdu, China; d General Ward 2, Sichuan Cancer Hospital, Chengdu, China.

**Keywords:** bibliometric analysis, coronary heart disease, exercise rehabilitation, research hotspots, research trends

## Abstract

Exercise rehabilitation can improve the prognosis of patients with coronary heart disease. However, a bibliometric analysis of the global exercise rehabilitation for coronary heart disease (CHD) research topic is lacking. This study investigated the development trends and research hotspots in the field of coronary heart disease and exercise rehabilitation. CiteSpace software was used to analyze the literature on exercise therapy for CHD in the Web of Science Core Collection database. We analyzed the data of countries/institutions, journals, authors, keywords, and cited references. A total of 3485 peer-reviewed papers were found, and the number of publications on the topic has steadily increased. The most productive country is the USA (1125), followed by China (477) and England (399). The top 3 active academic institutions are Research Libraries UK (RLUK) (236), Harvard University (152), and the University of California System (118). The most commonly cited journals are *Circulation* (2596), The most commonly cited references are “Exercise-based cardiac rehabilitation for coronary heart disease” (75), Lavie CJ had published the most papers (48). World Health Organization was the most influential author (334 citations). The research frontier trends in this field are body composition, participation, and function. Research on the effects of physical activity or exercise on patients with CHD is a focus of continuous exploration in this field. This study provides a new scientific perspective for exercise rehabilitation and CHD research and gives researchers valuable information for detecting the current research status, hotspots, and emerging trends for further research.

## 1. Introduction

Coronary heart disease (CHD) is the most common cause of death worldwide.^[1]^ This common cardiovascular disease presents with myocardial ischemia, hypoxia and even necrosis caused by coronary atherosclerosis, seriously threatening human life and health. In recent years, the incidence of CHD has increased rapidly and become an important public health problem.^[2]^ Given the broad impact of CHD in the public health field, effective therapeutic intervention for the condition has become a popular topic in medical research.

Percutaneous coronary intervention (PCI) has become one of the important methods of myocardial reperfusion in patients with coronary heart disease due to its advantages of high safety, little trauma, and rapid recovery.^[3]^ However, postoperative patients still face the risk of intrastent restenosis and intrastent thrombosis,^[4]^ which greatly weakens the clinical benefits of PCI. Exercise rehabilitation can reduce the risk of cardiovascular events in patients after PCI, and its effect is independent of drug therapy and nutritional intervention.^[5]^Exercise rehabilitation improves VO2 Max and endurance, or the ability to maintain physical activity for a long period of time.^[6]^ Exercise rehabilitation has a variety of other potential beneficial effects, including improved endothelial function, myocardial blood flow reserve, reduced smoking, weight loss, and lower lipids and blood pressure.^[7–9]^ Exercise rehabilitation can also reduce depression and anxiety in patients with heart disease and improve their quality of life.^[10]^The future of sports rehabilitation faces both opportunities and obstacles. Existing data and guidelines strongly support the role of comprehensive exercise rehabilitation in patients with CHD. Patients benefit from increased mortality, morbidity, disability and quality of life. Despite this, cardiac rehabilitation has clear benefits for patients with CHD, but referral and participation rates remain low. Telemedicine is able to expand the scope of rehabilitation services, and clinicians are unable to have face-to-face contact with patients, which can lead to patients not being able to properly complete rehabilitation exercise programs.

Our primary goal was to understand the status of research and participation in exercise rehabilitation for coronary heart disease since 2013; The second goal is to analyze the research trends and frontiers in this field to provide a scientific basis for better application of exercise rehabilitation in patients with coronary heart disease in the future. In this study, the literature on coronary heart disease exercise rehabilitation in Web of Science database was analyzed by CiteSpace,hoping to provide a basis for further research in this field in the future.

## 2. Methods

### 2.1. Data collection

**Figure 1.**
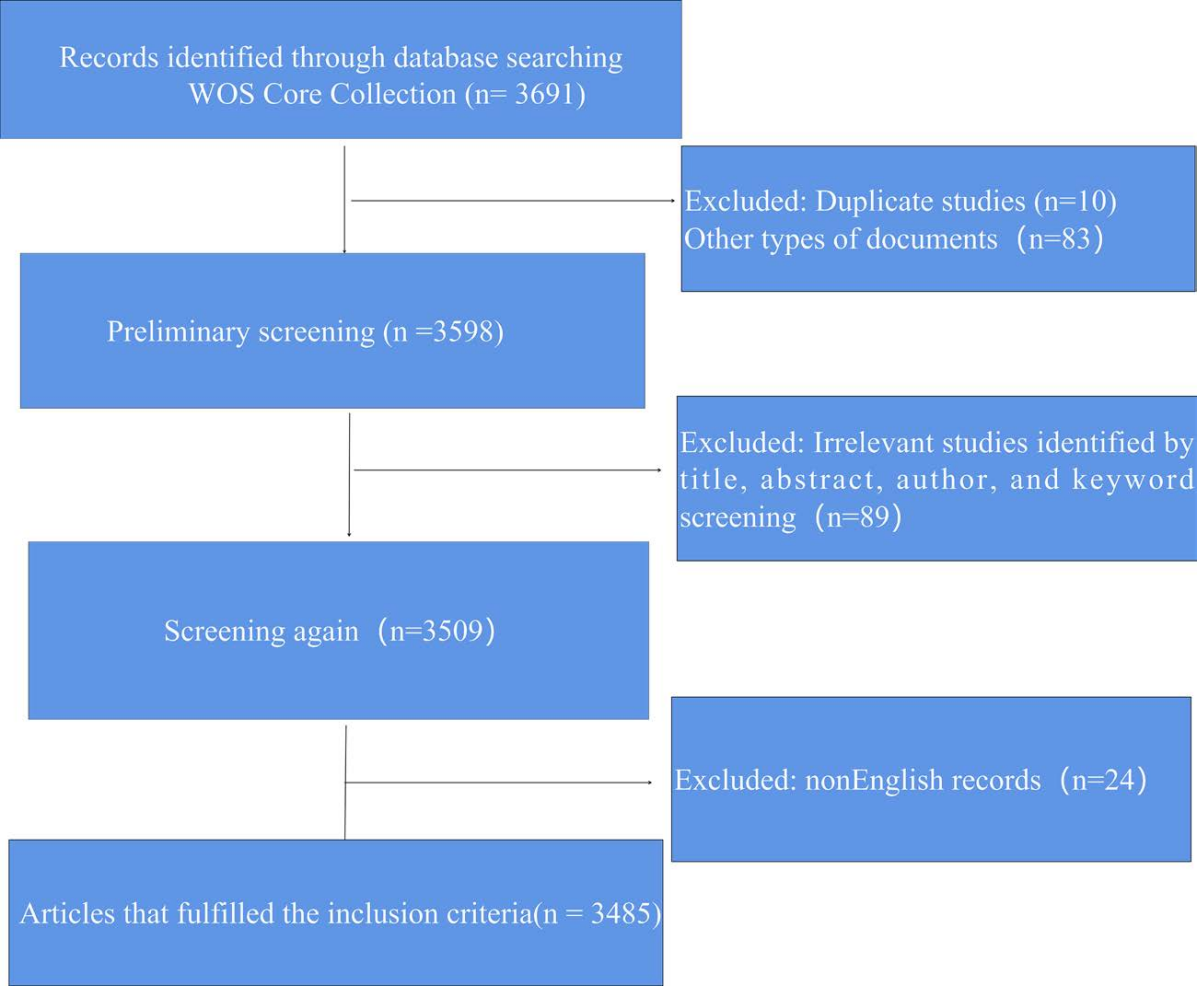
Flow chart of data collection.

This study used the WOSCC database developed by the American Institute of Scientific Information as the source of data to ensure the integrity and accuracy of the data obtained. Data was collected on “exercise” or “exercise therapy” or “training” or “physical activity” or “sport” or “fitness” or “walk “or “run” or “swim” or “jog “or “cycling” or “pilates” or “yoga” or “qigong” or “Tai Ji” and “coronary heart disease” or “CHD” in English from 2013.01 to 2023.05. The file types included “article” and “review.” A total of 3691 articles were retrieved, and after excluding invalid or non-English or duplicate articles and articles not related to the subject, the final number of included studies was 3485. The specific retrieval process is detailed in Figure 1.

### 2.2. Research methods

Version CiteSpace 6.6. R2 is a Java-based paper bibliometric analysis application involving the analysis of national institutions, journals, authors, references, and keywords. CiteSpace The parameters of the software were set as follows: the number of years per slice = 1, node type = once the appropriate analysis target was selected, and pruning = pathfinder. CiteSpace captures the research trends in coronary motor rehabilitation, explores the key paths and turning points in the development of the field, and forms a series of visual maps to explore the development frontiers of the field.

## 3. Results

### 3.1. Global publication trends

**Figure 2.**
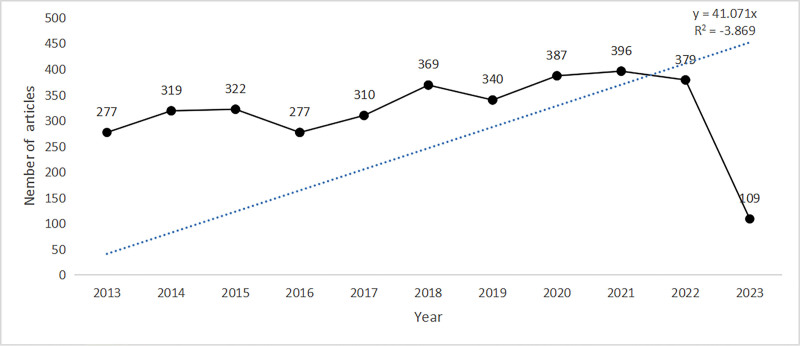
Annual distribution of publications in the field of exercise rehabilitation for CHD. CHD = coronary heart disease.

Data from the 3485 publications in the WOS core collection database were used for published statistics worldwide. Global release trends can illustrate the trends in a research hotspot, which are represented by line charts (Fig. 2). From 2013 to 2018, the number of global publications increased from 277 to 369, an increase of nearly 33%. Then, this number dropped slightly in 2019 but peaked in 2021, with 396 publications. The figure decreased slightly in 2022. Although the number of publications fluctuated in some periods, the overall yield was increasing.

### 3.2. National/Institutional visualizations

**Figure 3.**
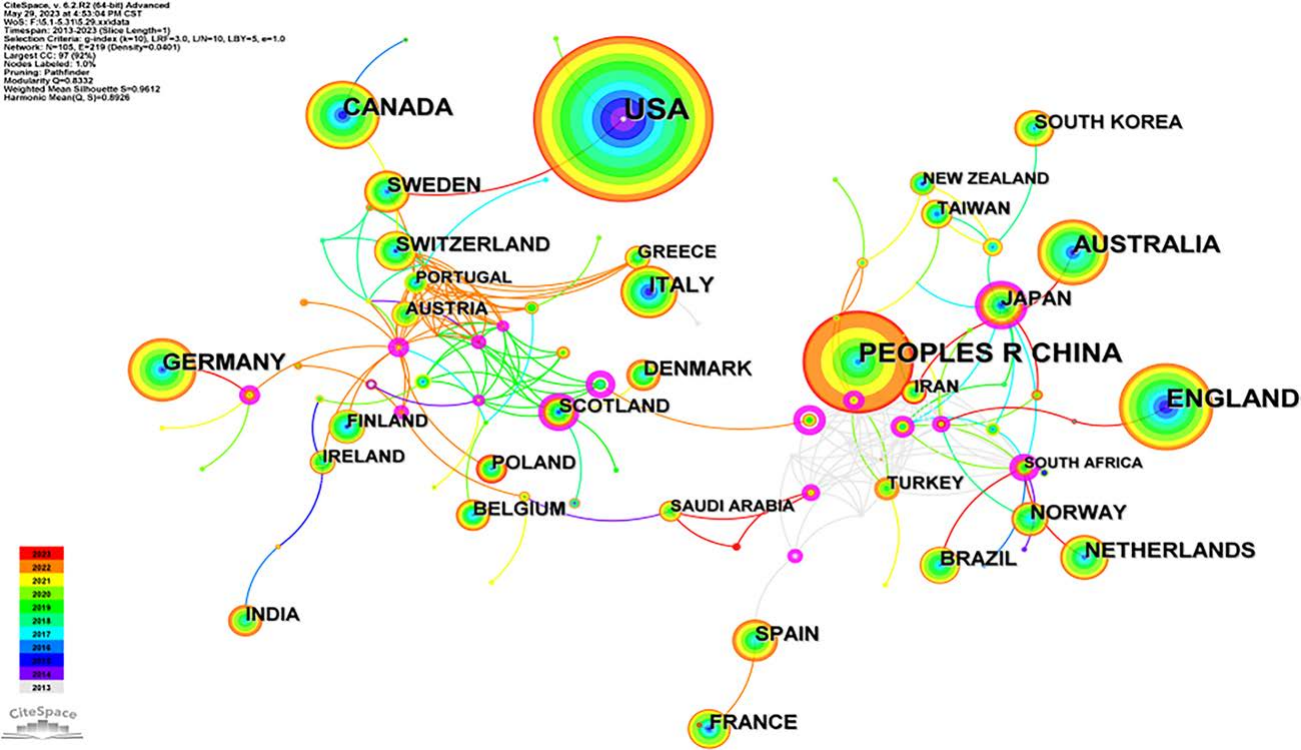
Network of relationships between publishing countries in the field of exercise rehabilitation for CHD. CHD = coronary heart disease.

A total of 3485 papers in the field of CHD exercise rehabilitation were retrieved, spanning 105 countries and 234 institutions. Table 1 lists the top 10 countries and institutions. The United States had the largest number of publications (1125), making it the most critical country in the field. Of the top 10 institutions, 3 were in the US, which could explain their larger proportion of total papers, followed by China (477) and the UK (399). Purple rings reflect centrality, and countries with high centrality are known as key points in the publications. The country with the highest centrality was Japan (0.12), followed by Sweden (0.07), and the United States, Canada and Italy (0.04) tied for third place. As shown in Figure 3, there was close cooperation among different countries. The most productive institution was the RLUK-British research library (236), followed by Harvard University (152), the University of California system (118), Harvard Medical School (100) and the University of London (97). Considering the number of papers published by research institutions, they could be considered the main institutions in the field of CHD exercise rehabilitation.

**Table 1:** Top 10 most productive countries/institutional areas in the field of exercise rehabilitation for coronary heart disease.

Rank	Country	Publications	Centrality	Institution	Publications	Centrality
1	USA	1125	0.04	RLUK-Research Libraries UK	236	0.12
2	CHINA	477	0	Harvard University	152	0.07
3	ENGLAND	399	0	University of California System	118	0.3
4	CANADA	259	0.04	Harvard Medical School	100	0.08
5	AUSTRALIA	258	0	University of London	97	0.07
6	GERMANY	232	0	University of Toronto	74	0.11
7	NETHERLANDS	145	0	Brigham & Women Hospital	73	0.16
8	ITALY	141	0.04	University of Sydney	72	0.24
9	JAPAN	123	0.12	University College London	67	0
10	SWEDEN	114	0.07	N8 Research Partnership	64	0.12

### 3.3. Journal co-citations

Table 2 lists the common citations and centrality ranking of academic journals in the field of exercise rehabilitation for CHD. The top 5 co-cited journals were CIRCULATION (2596), J AM COLL CARDIOL (1727), JAMA-JOURNAL OF THE AMERICAN MEDICAL ASSOCIATION (1664), LANCET (1532), and NEW ENGLAND JOURNAL OF MEDICINE (1476). Among the top ten co-cited journals, the journal with the highest impact factor was LANCET (IF 202.731). The most centrality journal was JAMA-JOURNAL OF THE AMERICAN MEDICAL ASSOCIATION, which belongs to Medical Region 1 articles with an IF of 157.335.

**Table 2:** Top ten active co-cited journals in the field of exercise rehabilitation for coronary heart disease.

Rank	Cited journal	Cocitation counts	Rank	Cited journal	Centrality
1	CIRCULATION	2596	1	JAMA-JOURNAL OF THE AMERICAN MEDICAL ASSOCIATION	1.01
2	JOURNAL OF THE AMERICAN COLLEGE OF CARDIOLOGY	1727	2	CIRCULATION	0.95
3	JAMA-JOURNAL OF THE AMERICAN MEDICAL ASSOCIATION	1664	3	EUROPEAN JOURNAL OF CARDIOVASCULAR PREVENTION & REHABILITATION	0.64
4	LANCET	1532	4	DIABETES CARE	0.64
5	NEW ENGLAND JOURNAL OF MEDICINE	1476	5	DIABETOLOGIA	0.61
6	EUROPEAN HEART JOURNAL	1418	6	JOURNAL OF LIPID RESEARCH	0.60
7	AMERICAN JOURNAL OF CARDIOLOGY	1205	7	JOURNAL OF CLINICAL ENDOCRINOLOGY & METABOLISM	0.58
8	PLOS ONE	1160	8	JOURNAL OF CARDIOPULMONARY REHABILITATION AND PREVENTION	0.53
9	MEDICINE& SCIENCE IN SPORTS & EXERCISE	1115	9	MEDICINE& SCIENCE IN SPORTS & EXERCISE	0.36
10	INTERNATIONAL JOURNAL OF CARDIOLOGY	989	10	JOURNAL OF CLINICAL INVESTIGATION	0.35

### 3.4. Co-cited references and cluster analysis

**Figure 4.**
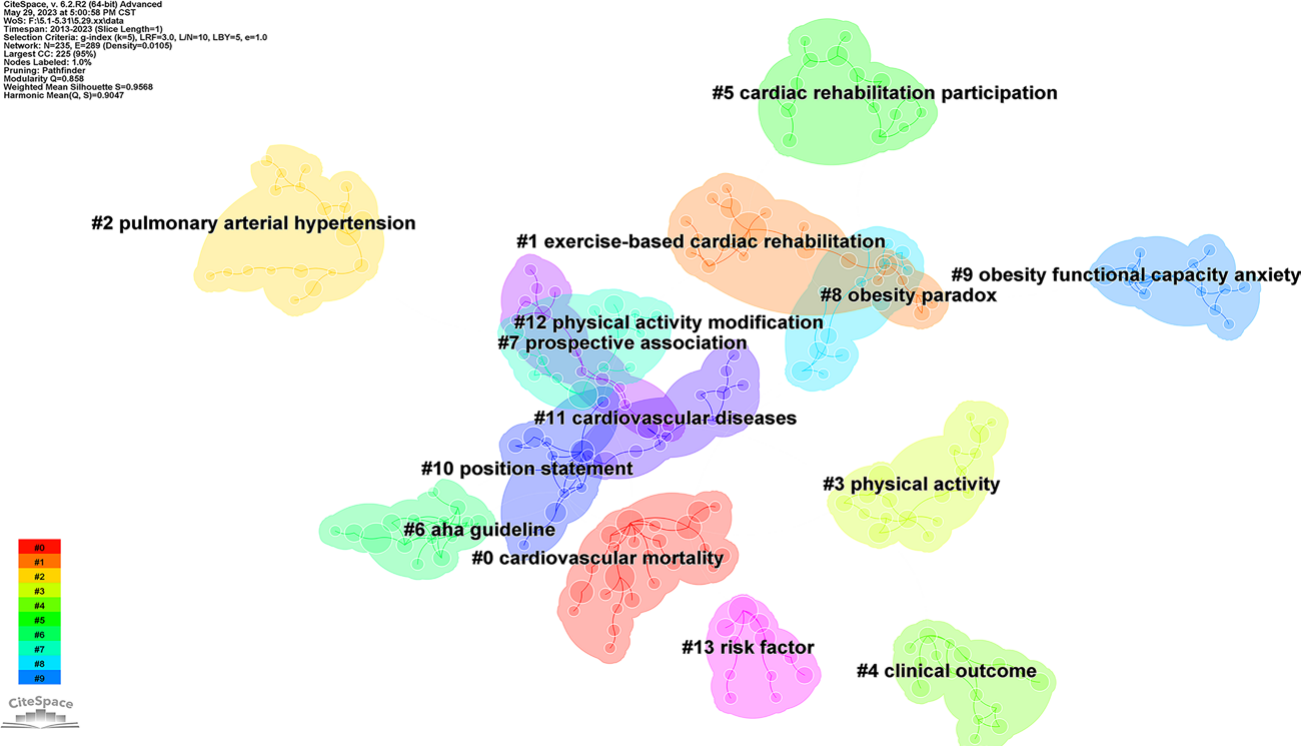
The cluster diagram of co-cited references in the field of exercise rehabilitation for CHD. CHD = coronary heart disease.

Co-referenced references refer to 2 or more papers cited by one or more papers at the same time. Over the past 10 years, 235 co-cited references in the field of exercise rehabilitation for CHD were identified. The total number of references was 2453. Table 3 shows that the most cited articles were from the Cochrane Systematic Review Database, with a frequency of 75 citations. This article was written by lead author Balraj S Heran and was published in 2011. Of the 10 most cited articles, 2 were from CIRCULATION. The cluster analysis of the literature co-citations using the CiteSpace clustering function (Fig. 4) identified 14 common themes similar to the literature. According to the number of nodes, the top 5 themes were cardiovascular mortality, exercise-based cardiac rehabilitation, pulmonary hypertension, physical activity, and clinical outcomes.

**Table 3:** Top ten most co-cited references in the field of exercise rehabilitation for coronary heart disease.

Rank	Title	Author	Periodicals	Frequency of Citations	Yr
1	Exercise-based cardiac rehabilitation for coronary heart disease	Balraj S Heran	COCHRANE DATABASE OF SYSTEMATIC REVIEW	75	2011
2	Heart Disease and Stroke Statistics-2018 Update: A Report From the American Heart Association	Emelia J Benjamin	CIRCULATION	52	2018
3	2016 European Guidelines on cardiovascular disease prevention in clinical practice: The Sixth Joint Task Force of the European Society of Cardiology and Other Societies on Cardiovascular Disease Prevention in Clinical Practice (constituted by representatives of 10 societies and by invited experts)Developed with the special contribution of the European Association for Cardiovascular Prevention & Rehabilitation (EACPR)	Massimo F Piepoli	EUROPEAN HEART JOURNAL	49	2016
4	The Physical Activity Guidelines for Americans	Katrina L Piercy	JAMA Surgery	46	2018
5	Dose response between physical activity and risk of coronary heart disease: a meta-analysis	Jacob Sattelmair	CIRCULATION	45	2011
6	Leisure time physical activity and mortality: a detailed pooled analysis of the dose–response relationship	Hannah Arem	AMA INTERN MED	41	2015
7	Importance of Assessing Cardiorespiratory Fitness in Clinical Practice: A Case for Fitness as a Clinical Vital Sign: A Scientific Statement From the American Heart Association	Robert Ross	CIRCULATION	36	2016
8	2015 ESC/ERS Guidelines for the diagnosis and treatment of pulmonary hypertension: The Joint Task Force for the Diagnosis and Treatment of Pulmonary Hypertension of the European Society of Cardiology (ESC) and the European Respiratory Society (ERS): Endorsed by: Association for European Pediatric and Congenital Cardiology (AEPC), International Society for Heart and Lung Transplantation (ISHLT)	Nazzareno Galiè	EUROPEAN HEART JOURNAL	35	2016
9	Leisure-time running reduces all-cause and cardiovascular mortality risk	ploDuck-Chul Lee	JOURNAL OF THE AMERICAN COLLEGE OF CARDIOLOGY	35	2014
10	2016 European Guidelines on cardiovascular disease prevention in clinical practice: The Sixth Joint Task Force of the European Society of Cardiology and Other Societies on Cardiovascular Disease Prevention in Clinical Practice (constituted by representatives of 10 societies and by invited experts) Developed with the special contribution of the European Association for Cardiovascular Prevention & Rehabilitation	Massimo F Piepoli	ATHEROSCLEROSIS	32	2016

### 3.5. Author and co-cited author analysis

**Figure 5.**
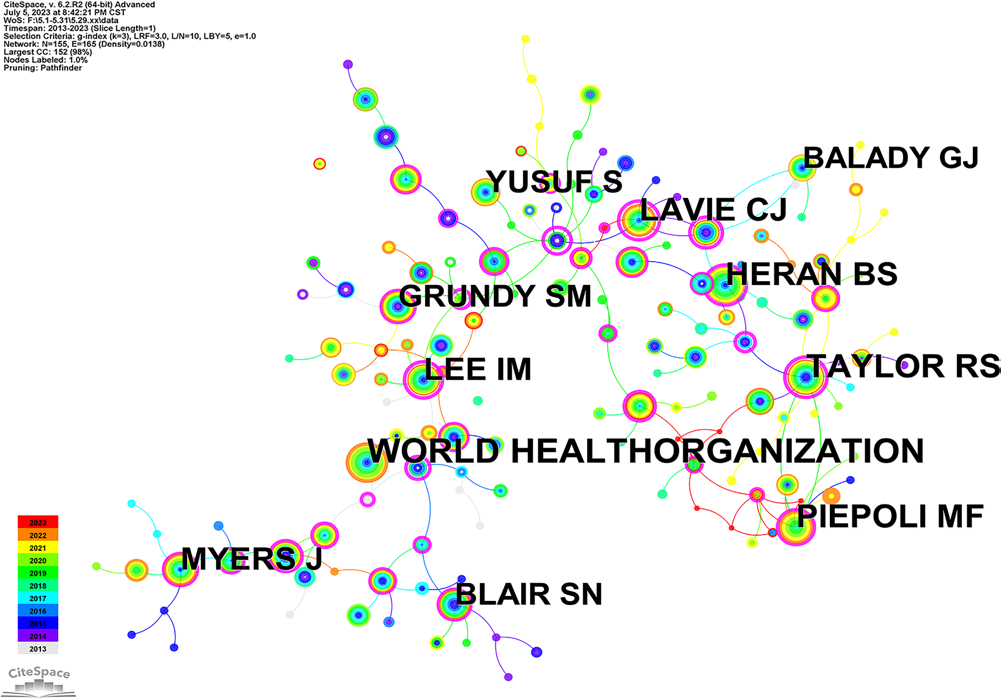
The network of co-cited authors in the field of exercise rehabilitation for CHD. CHD = coronary heart disease.

To fully exploit the influential authors in the field and show the cooperative network between the authors, we drew a map of the cited authors with CiteSpace software (Fig. 5). A total of 3485 papers had contributions from 510 authors. Table 4 lists the top 10 authors by number of papers; the top 5 authors are Lavie, Carl J; Ewert, Peter; Arena, Ross; Mueller, Jan and Hager, Alfred. However, these authors all had centrality values of <0.1, indicating a low intensity of collaboration between researchers. As seen from Figure 5, the top 3 authors were the World Health Organization (considered a group author; 334 citations), Taylor, Rod S (250 citations) and Lavie, Carl J (244 citations); of these, Lavie, Carl J had a higher comprehensive ability than other authors, and his higher individual citations and h-index make him a high academic influence.

**Table 4:** Top ten active authors in the field of exercise rehabilitation for coronary heart disease.

Author	Published articles	Average per item citations	H-index	Country	Centrality
Lavie, Carl J	48	36.96	98	USA	0.02
Ewert, Peter	24	10.54	25	Germany	0
Arena, Ross	23	15.27	20	USA	0.04
Mueller, Jan	18	23.04	20	Germany	0
Hager, Alfred	17	18.51	31	Germany	0
Laukkanen, Jari A	15	6.1	9	Finland	0
Oberhoffer, Renate	14	12.08	22	Germany	0
Milani, Richard V	14	38.64	67	USA	0.01
Blumenthal, James A	13	68.7	87	USA	0.02
Kunutsor, Setor K	12	25.48	45	Britain	0

### 3.6. Keyword analysis

#### 3.6.1. Keyword co-occurrence.

**Figure 6.**
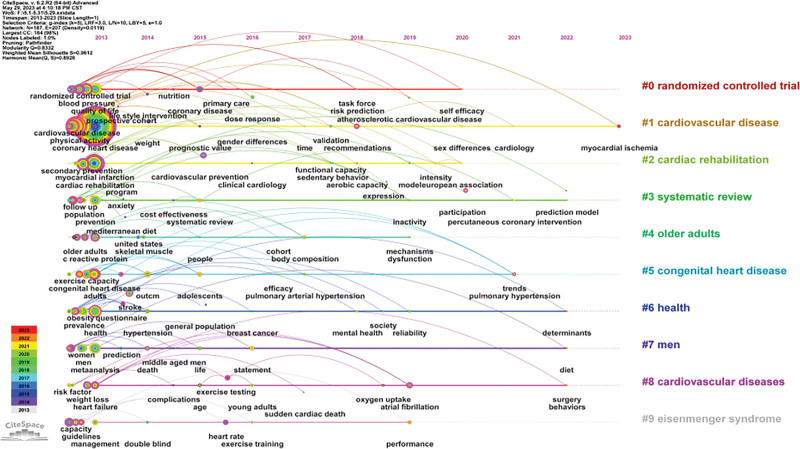
Timeline view map of the keywords in the field of exercise rehabilitation for CHD. CHD = coronary heart disease.

A keyword in academic research reflects not only the focus in a certain research field but also the research heat and research direction in that field. In this study, 187 keywords were identified, with a total frequency of 16827. Figure 6 shows the map of keyword co-occurrence in the last 10 years, indicating the scope of research in the field of exercise rehabilitation for CHD. As shown in Table 5, after excluding search terms and meanings similar to coronary heart disease, cardiovascular disease, exercise, and cardiac rehabilitation, the highest frequency keywords were “physical activity,” “risk,” “mortality,” “risk factors” and “association,” while “coronary heart disease,” “adherence,” “randomized controlled trial” and “cardiac rehabilitation” had the highest centrality.

**Table 5:** Top ten high-frequency keywords in the field of exercise rehabilitation for coronary heart disease.

Rank	Keyword	Frequency	Keyword	Centrality
1	Coronary heart disease	2100	Coronary heart disease	0.56
2	Physical activity	915	Adherence	0.48
3	Cardiovascular disease	687	Randomized controlled trial	0.48
4	Exercise	635	Cardiac rehabilitation	0.46
5	Risk	523	Myocardial infarction	0.42
6	Mortality	455	Coronary disease	0.39
7	Risk factors	446	Secondary prevention	0.38
8	Cardiac rehabilitation	392	Weight loss	0.37
9	Myocardial infarction	383	Guidelines	0.37
10	Association	340	Atrial fibrillation	0.37

#### 3.6.2. Keyword timeline.

On the basis of the keyword co-occurrence analysis, to better analyze the popular topics and trends in the field of coronary heart disease sports rehabilitation, keyword clustering timeline analysis was conducted (Fig. 6). The output clustering results from the keyword cluster table (Table 6) reveals that among the 10 group-related cluster labels, 5 of the largest clusters were #0: randomized controlled trial, #1: cardiovascular disease, #2: cardiac rehabilitation, #3: systematic review, and #4: older adults. Physical activity had the largest node, indicating that the word is a research hotspot in this field. Additionally, the 5 groups with the highest S value were # 4: older adults, #5: congenital heart disease, # 8: cardiovascular disease, #6: health and # 2: cardiac rehabilitation.

**Table 6:** Network map of keyword clusters in the field of exercise rehabilitation for coronary heart disease.

Rank	Size	Silhouette	Cluster	Keywords (partial)
#0	21	0.97	Randomized controlled trial	Quality of life; life style; blood pressure; humans
#1	21	0.917	Cardiovascular disease	Physical activity; congenital heart disease; quality of life; risk
#2	19	0.973	Cardiac rehabilitation	Secondary prevention; myocardial infarction; acute myocardial infarction; coronary artery disease
#3	16	0.961	Systematic review	Predictors; anxiety; metabolic syndrome; mediterranean diet
#4	15	0.983	Older adults	Inflammation; skeletal muscle; c-reactive protein; cardiovascular risk factors
#5	15	1	Congenital heart disease	Exercise capacity; pulmonary arterial hypertension; children; pulmonary hypertension
#6	15	0.98	Health	Prevalence; validity; reproducibility; care
#7	14	0.961	Men	Leisure time; women; meta-analysis; congenital heart disease
#8	13	1	Cardiovascular diseases	Heart failure; weight loss; risk factor; cardiac rehabilitatio
#9	9	0.902	Eisenmenger syndrome	Management; pulmonary arterial hypertension; congenital heart disease; pulmonary hypertension

#### 3.6.3. Keyword time zone map.

**Figure 7.**
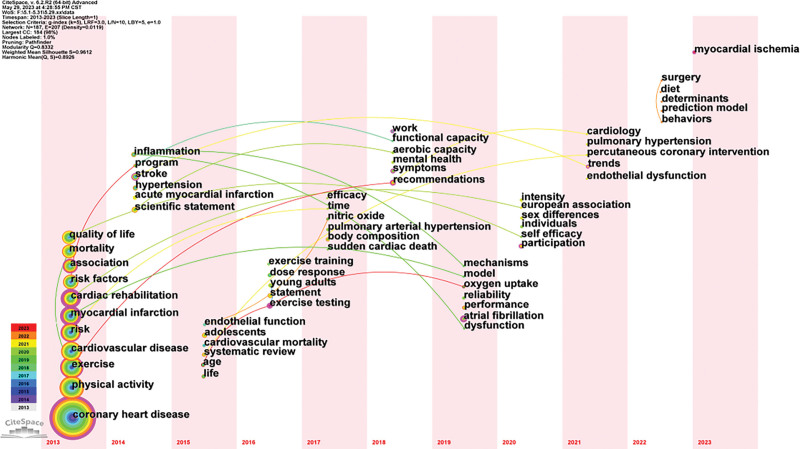
Keyword time-zone map in the field of exercise rehabilitation for CHD. CHD = coronary heart disease.

The time zone map is composed of a series of vertical zones of time. It mainly shows the knowledge graph of the evolution of keywords from the time dimension and is capable of not only clearly showing the relationship among keywords but also how keywords are updated. As shown in the time zone map (Fig. 7), the hotspots changed from “coronary heart disease,” “physical activity,” “risk,” “exercise,” “cardiac rehabilitation,” “myocardial infarction,” “association,” and “mortality” to “myocardial,” “ischemia,” “surgery,” “dish,” “determinants,” “prediction model,” “behaviors,” “cardiology,” and “pulmonary hypertension.” Due to the rapid development of big data, medical treatment has also shifted from simple techniques to teams of medical staff, with more emphasis on early prediction and precise intervention.

#### 3.6.4. Keyword citation bursts.

**Figure 8.**
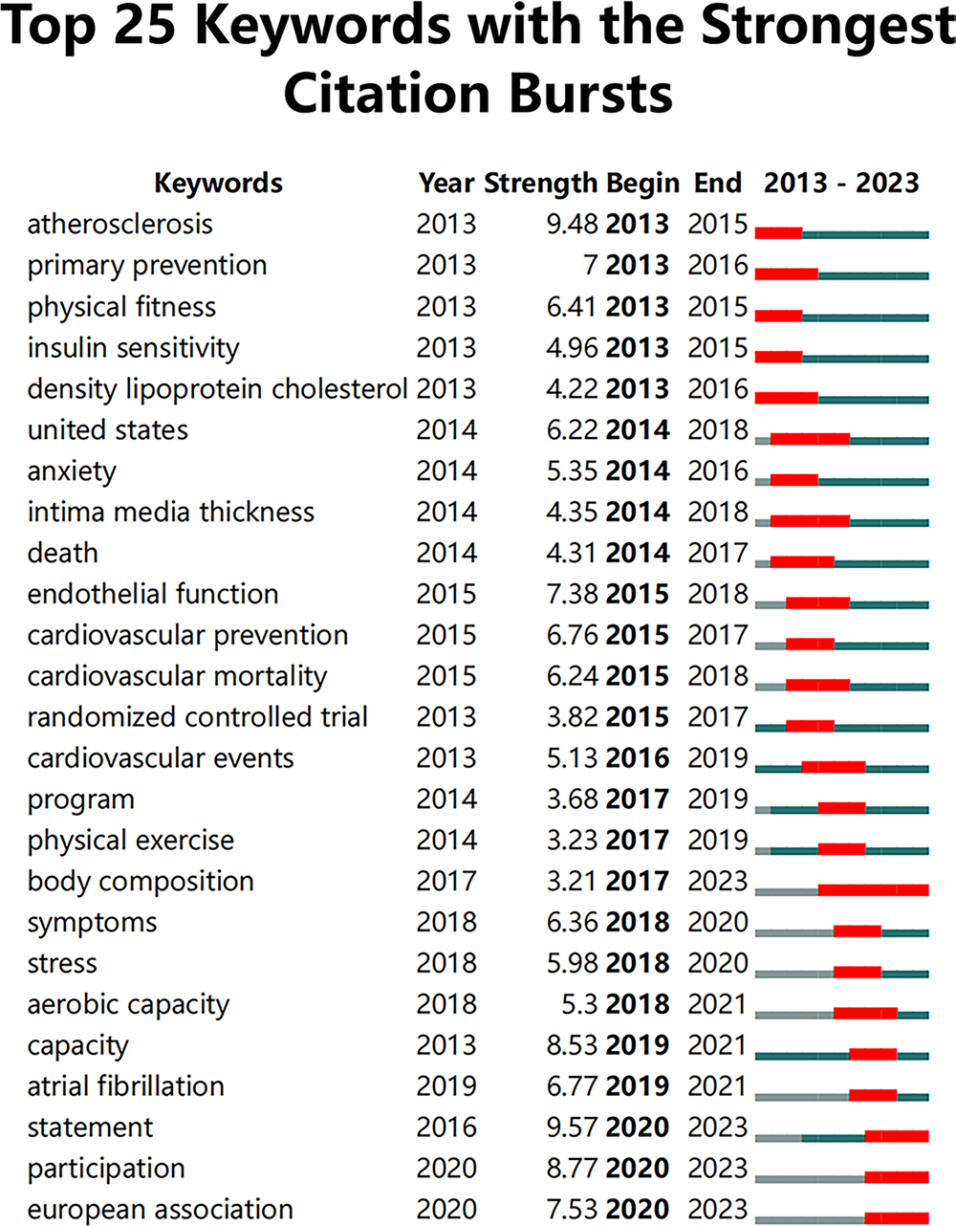
The top twenty-five keywords with the strongest citation bursts in the field of exercise rehabilitation for CHD. CHD = coronary heart disease.

CiteSpace can show how research hotspots change with time to reflect the dynamics of a research field and the changes in popularity in particular research directions to a certain extent. Setting the number of years of each fragment to 1 gives the keyword outbreak map in coronary heart disease exercise rehabilitation over the past 10 years (Fig. 8). The key words of the recent outbreak were “declaration,” “participation” and “European association.” The European Association refers to the European Association for the Prevention of Cardiology, which published in 2020 an update on the core content and goals of cardiac rehabilitation, highlighting the use of different integrated rehabilitation strategies for challenging populations and practical recommendations for different exercise training regimens.^[11]^ The American Heart Association has issued specific recommendations for physical activity in healthy people and people with different chronic diseases,^[12]^ and exercise participation was identified a focus in the field of exercise rehabilitation for coronary heart disease.

## 4. Discussion

### 4.1. General information on exercise rehabilitation for coronary heart disease

In the past decade, the field of exercise rehabilitation for coronary heart disease has received extensive attention. This phenomenon is because exercise interventions reduce cardiovascular mortality and hospitalization risk, and improve exercise capacity and health-related quality of life in patients with CHD (i.e., after myocardial infarction, after revascularization, and angina).^[13]^ International clinical guidelines recommend exercise rehabilitation as a core component of cardiac rehabilitation^[14]^ and suggest that cardiac rehabilitation based on exercise rehabilitation is effective at all stages of cardiac rehabilitation. CiteSpace is now widely used to study subject dynamics, visualize the structure, patterns and distribution of scientific knowledge, and summarize the relationships between countries, institutions, authors and journals.

According to the analysis of national and institutional networks, the United States is the leading country in CHD and sports rehabilitation research, with the largest number of papers published (1125). Among them, China performs well in the number of published papers (477), but it has no advantage in centrality, indicating that the influence in the study is still insufficient. Nine out of the top 10 countries are developed countries, and only China is a developing country. In this research area, there is still a considerable gap between developing and developed countries. Among them, the country with the highest centrality was Japan, indicating that it has had a greater influence in this field. The institutions were primarily the UK Research Library, Harvard University, the University of Toronto in Canada and the University of Sydney in Australia. The top 10 institutions accounted for 25% of the total publication rate, meaning that they have achieved substantial academic achievements. However, research collaborations are mainly conducted between neighboring countries and are not sufficiently dispersed.

By analyzing the co-cited journals on exercise rehabilitation and CHD, we learned that the researchers focus on 2 disciplines: cardiovascular system and sports medicine. Among the journals, CIRCULATION (2596) and MEDICINE & SCIENCE IN SPORTS & EXERCISE (1115) contributed the most to these 2 disciplines, indicating that the research field has been focused on internal sciences and kinematics. The influence of the top 5 cited journals was 23.7, among which LANCET had the highest impact factor (202.731), indicating that the field of exercise rehabilitation for coronary heart disease received high investment and attention from high-quality journals.

The author contribution analysis (Table 4) shows that Lavie, Carl J from the US ranked first in the number of published articles, indicating that their team has contributed and invested heavily in the field. An analysis of the author co-citation chart (Fig. 5) shows that Lavie, Carl J has been cited 244 times, indicating that his research results have been recognized by many scholars. Lavie, Carl J research interests include cardiac rehabilitation and prevention, blood lipids, hypertension, obesity and exercise, and their studies mainly examine the effects of sedentary behavior and physical activity on patients with cardiovascular disease,^[15–17]^ especially in patients with heart failure and reduced ejection fraction.^[18,19]^ Their research also compares various effective exercise methods, and the advantages and limitations of resistance training and moderate intensity aerobic training are summarized to find the best form of exercise for patients with heart disease.^[20,21]^ Studies on the effects of moderate and vigorous physical activity (MVPA) and sedentary behavior (SB) on life expectancy in patients with heart failure (HF) have shown that excessive SB impairs the beneficial effects of MVPA on heart disease.^[22]^ In addition, Lavie, Carl J outlines the benefits of exercise-based cardiac rehabilitation for cardiorespiratory fitness, providing constructive advice on exercise for patients with CHD, and it states that in prescription for CAD, exercise training is still inadequate.^[23]^

Of the 10 most cited articles, “exercise-based CHD cardiac rehabilitation” was the most co-cited (75), indicating a high academic value. This study compares the impact of exercise-based cardiac rehabilitation with usual care in patients with CHD. The results showed that exercise-based cardiac rehabilitation can effectively reduce the overall mortality, cardiovascular mortality and hospitalization rates in CHD patients,^[24]^ providing retrospective and evidence-based evidence for exercise-based cardiac rehabilitation in CHD patients. Adaptive exercise prescriptions should be given to all eligible CHD patients to control risk factors and reduce medical costs. CHD patients are encouraged to perform regular exercise rehabilitation to achieve maximum benefit.

### 4.2. Research hotspots in the field of exercise rehabilitation of CHD

Based on co-cited keywords and co-cited references, we identified the following hotspots in exercise rehabilitation for CHD. Research on the effects of physical activity or exercise on patients with CHD is a focus of continuous exploration in this field. It is well known that common risk factors for coronary heart disease include smoking, hypertension, abnormal lipid levels, obesity and diabetes. Exercise training is currently an important part of CHD management. Regular exercise can reduce the damage of cardiovascular risk factors to patients, improve exercise tolerance in patients with coronary heart disease, and improve cardiovascular function,^[25]^ thus reducing cardiovascular mortality.^[1]^ Studies have shown that after 3 to 6 months of exercise training, the peak value of vanadium dioxide increased by 11% to 36%, and the worse the condition of patients, the more obvious the improvement effect was.^[26]^ Exercise training can increase the diameter and elasticity of coronary arteries, improve endothelial function, increase the coronary blood supply, and stabilize coronary artery clots, thus improving cardiac function in patients with CHD.^[20]^ Long-term regular aerobic exercise can also reduce systolic blood pressure (SBP) and better control the complications of hypertension.^[27]^The effect of exercise also depends largely on the intensity of endurance during exercise. One study found that the survival rate in patients with an exercise endurance <10 MET was significantly lower than that in patients with an exercise endurance >18 MET.^[28]^ While improving physical activity endurance and quality of life, people with CHD can also independently participate in daily activities, which is crucial to improving the psychology of elderly individuals and relieve stress within the family and in society as a whole. The practical significance of exercise rehabilitation training for CHD patients has been confirmed by strong studies. Clinical studies have shown that exercise-based cardiac rehabilitation (CR) is effective in reducing all-cause mortality by 8% to 37% after myocardial infarction (MI),^[29]^ as well as in reducing morbidity and hospitalization related to acute ischemic coronary events. In recent years, the neutrophil-to-lymphocyte ratio (NLR) has become a novel inflammatory marker for identifying coronary artery disease (CAD),^[30]^which predicts the clinical outcome in patients with CHD.^[31]^ Many researchers are interested in the relationship between exercise rehabilitation training and systemic inflammation in CHD patients, and some studies suggest that exercise-based CR programs improve the neutrophil/lymphocyte ratio (N/L) in patients with CAD.^[32]^ The effect of different exercise types on CHD patients is another hotspot in this field. Acupuncture combined with aerobic exercise has a positive impact on cardiopulmonary capacity, blood lipids, fatty acid oxidation and psychology in CHD patients,^[33]^ while extracorporeal anti-bo combined with high-intensity aerobic exercise significantly reduced the incidence of major postoperative adverse cardiovascular events and postoperative activities in PCI patients and improved their daily living ability.^[34]^ Regarding the most effective types of exercise, studies have shown that a combination of aerobic, resistance, breathing or inspiratory muscle training has the best results in improving aerobic capacity, muscle strength, functional capacity, ventilatory response and quality of life in cardiovascular patients.^[35]^ The influence of different exercise management models on CHD patients is yet another research hotspot in this field. In a randomized controlled study, multidisciplinary exercise management based on mobile apps had high effectiveness in patients undergoing PCI, effectively improving motor compliance, disease-related cognition, self-efficacy, and social support perception after the intervention.^[36]^

### 4.3. Global trends in the field of exercise rehabilitation for CHD

Keyword burst can summarize the keywords showing high frequency changes to reveal the research frontiers in the development of sports rehabilitation regimens for coronary heart disease. According to our comprehensive analysis of keyword emergence, the following conclusions can be made regarding emerging trends in exercise rehabilitation for coronary heart disease:

#### 4.3.1. Body composition.

Exercise training is associated with improved blood lipid and body fat composition in patients with CHD.^[37]^ Body composition includes fat mass in the body, body moisture, and muscle mass.^[38]^ With continuous study of heart disease-related sarcopenia, the relationship between body composition and cardiovascular disease has become increasingly valued by researchers. Body composition can be measured by bioimpedance, quantitative magnetic resonance, air-displacement plethysmography, and magnetic resonance imaging.^[39]^ Body composition reports have shown that an increase in the fat-free mass is directly associated with increased muscle strength. A recent systematic evaluation showed that aerobic exercise training for 12 weeks or more in patients with metabolic syndrome improved body composition, glucose and lipid metabolism, and physical fitness in patients with metabolic syndrome.^[40]^ When the aerobic exercise intensity reached 75 to 90% of the maximum oxygen consumption (vo2max), high- and low-density lipoprotein cholesterol (Ldl-c) and total cholesterol (Tc) were reduced to a certain degree,^[41,42]^ and high-density lipoprotein cholesterol (Hdl-c) and triglycerides (Tg) had a better dose response to the intensity and duration of aerobic exercise.^[43]^ Therefore, performing continuous, more intense aerobic exercise can improve patient body composition. It is well known that waist circumference is clinically a common indicator of diagnostic central obesity, with a 1 cm increased waist circumference associated with a 2% increased risk of cardiovascular events in men and a 5% increased risk of cardiovascular events in women.^[44]^ Chronic accumulation of body fat leads to a series of pathophysiological changes, such as increased cardiac output, inflammation, metabolic abnormalities, and atherosclerotic.^[45]^ These pathophysiological changes play an important role in organ damage. That is, overweight/ obese individuals have the development of organ damage that can lead to an increase in cardiac filling pressure and volume, thereby increasing cardiovascular work, leading to LV dilatation and hypertrophic.^[46]^ Recent studies showed that waist circumference may more accurately evaluate cardiac function through changes in left ventricular structure in patients undergoing precision cardiac rehabilitation than BMI.^[47]^ A potential mechanism to explain this association is that waist circumference represents central obesity, which affects cardiac structural and functional^[48–50]^ by increased cardiac load and hypoxia or ischemia in the cardiovascular system.It can lead to hypertension, arrhythmia, heart failure, etc^[48–50]^ which reflects the compensatory hypertrophy changes of left ventricular structure (left ventricular remodeling) caused by the increased extra load of the cardiovascular system.^[48–50]^ It has certain significance for the early diagnosis of coronary heart disease in obese patients, effective evaluation and appropriate adjustment of precise cardiac rehabilitation plan.

#### 4.3.2. Participation.

Despite progress in the safety of cardiac rehabilitation programs, only 15% to 30% of eligible patients worldwide participate.^[51,52]^ Even after participating in a cardiac rehabilitation (CR) program, only 50% of such participants had remained physically active at 6 months.^[53]^ Only by clarifying the factors affecting CHD patients’ participation in exercise rehabilitation can targeted intervention programs be developed to improve health outcomes. Among the many qualitative studies, Neubeck et al^[54]^ identified influencing factors such as service- and system-level barriers (doctor recommendations, medical interactions and misunderstandings of CR), practical barriers (traffic, cost and language), and personal barriers (views of disease and CR, disease control beliefs). Of these, the key factors were system-level and patient-level barriers, and the authors said that these barriers could be improved. Campkin et al^[55]^ conducted a literature analysis based on grounded theory, emphasizing the importance of social, cognitive and emotional factors in motor participation, which can be both hindering and facilitating factors. Positive or negative self-cognitive evaluation can modulate individual behavior. Once the patient cannot establish a positive concept of self-worth, they will terminate the exercise rehabilitation program. Moreover, center-based CR (CBCR) is the “gold standard,” but there are barriers to patient participation, including dislike of group exercise, geographic distance, and time inconvenience. Telerehabilitation guidance provided by telerehabilitation programs helps to develop patients’ self-efficacy and improve their motivation to participate in exercise rehabilitation.^[56]^ Family-based CR requests have increased in recent years due to the COVID-19 pandemic. Home-based cardiac telerehabilitation (HBCTR) demonstrated equivalent efficacy to CBCR^[57]^ and achieved significant efficacy over the medium to long term.^[58]^ A randomized controlled trial involving 49 CHD patients showed that 12 weeks of cardiac telerehabilitation training could effectively improve patients’ functional ability and improve their exercise participation.^[59]^ Cardiac telerehabilitation training patients had higher compliance than outpatient CR and traditional CR patients, which may be related to improved patient participation because patients flexibly adjust the time used for exercise rehabilitation, reducing the inconvenience caused by transportation. However, methods for ensuring that patients receive the appropriate exercise rehabilitation dose at home and effectively complete interventions remain unclear, and further exploration of such programs is necessary.

From the analysis of keyword strength, the emergence intensity of the keyword “capacity” in recent years was very high, reaching 8.53, indicating that improving the functional ability of patients may also be a research frontier in this field. Exercise capacity is considered an independent predictor of all-cause mortality and readmission in CR patients,^[60]^ and higher exercise capacity is associated with better clinical outcomes in patients with CHD. In terms of improving exercise performance, high-intensity interval training (HIIT) is more effective than medium-intensity continuous training (MICT) and can effectively improve the peak oxygen consumption and functional capacity of patients. Long-term and stronger HIIT significantly improves exercise endurance^[61]^ and has been shown to have fewer adverse effects in studies of heart failure.^[62]^ Performing HIIT requires a personalized exercise program according to the patient functional status and exercise preferences to ensure safe rehabilitation while reducing physical discomfort.

## 5. Conclusion

The findings of this bibliometric study can help researchers quickly find general information, research hotspots and research trends in the field of exercise rehabilitation for coronary heart disease from the past 10 years. In the current research landscape, the study of exercise and coronary heart disease still has great potential for development. The most influential country, institution, journal and author are the US, UK research libraries, CIRCULATION and Lavie, Carl J, respectively. “Body composition,” “participation” and “capacity” may be the latest research frontiers. Overall, this study provides new scientific perspectives for exercise and CHD research and provides valuable information for researchers, funding agencies, and policy makers.

### 5.1. Limitations

First, we only searched the literature in the Web of Science Core Collection database, and the analyses presented in this paper do not reflect the full context of the research area explored. Second, English was chosen as the language, which may lead to the omission of relevant studies published in other languages. Finally, this study could not include data for the full year of 2023, which prevented a comprehensive analysis of annually published statistics. Therefore, the reader should be aware that all these factors may lead to bias in our results.

## Acknowledgements

We thank the reviewers for allowing us to improve the manuscript.

## Author contributions

**Conceptualization:** Qing Wen, Qun-Hua Ma, Xiao-Li Tang.

**Formal analysis:** Lin-Zhang Li, Xue-Wu Song, Hu-Kui Han.

**Validation:** Lin-Zhang Li.

**Visualization:** Qing Wen, Qun-Hua Ma, Lin-Zhang Li, Xiao-Li Tang.

**Writing – original draft:** Qing Wen, Qun-Hua Ma, Xiao-Li Tang.

**Writing – review & editing:** Qing Wen, Qun-Hua Ma, Hu-Kui Han, Gui-Yu Huang, Xiao-Li Tang.
